# On the Operation of Cupping

**Published:** 1809-11

**Authors:** 


### *To the Editors of the Medical and Physical Journal.*

GENTLEMEN,

IN answer to the inquiries of your Correspondent on the subject of cupping, I freely communicate the successful experience of twenty years. The operation of cupping, though a very simple one, and needing but little or no anatomical knowledge, will be found to require more address and practice than is necessary for many of the major operations of surgery. When neatly performed, twelve or sixteen fluid ounces of blood may be obtained in half an hour, unless the surface to be operated on be very deficient in vascularity.

In

In all operations, so in this, it is necessary to have every requisite well arranged and at hand, as the moment the scarifications are made, the glasses should be fixed, and so again in removing and replacing them. Where the surface will allow it, the larger the glasses the better, not holding less than four or six ounces, the drawing powers being almost in proportion to their size; their shape should be that generally used, the bell. A scarificator, containing six good lancets, is more effectual than sixteen, as the resistance offered to a great number, prevents perfect incisions, so that frequently the surface only shall be scratched; even four lancets, if they perfectly enter the integuments, will be found, if the glass be well put on, to draw blood very readily.

There is some address, only to be learnt by practice, in applying the scarificator; a gentle yielding at the time the lancets are making their way through, will give more perfect incisions than when the instrument is too obstinately pressed against the part. It may be compared to the slight turn of the hand, which some operators give in extracting the cataract, when at the same time they scratch the capsule.

The lamp, if dexterously used, is certainly the neatest mode of rarifying the air, but few attain perfection in its use; hence, small bits of bibulous paper, dipped in spirits of wine, lighted, thrown into the glass, and the glass immediately fixed on the part, not suffering the paper to fall out, will be found the most certain method of exhausting the receivers.

On removing the glasses, the scarified parts are to be cleansed with sponge and warm water; the orifices to be well dried with a napkin, and a dry glass lighted as before, to be fixed on. Unless the operator is quick in his movements, it will be better to remove but one glass at a time, as the air tends to close the orifices.

Frequent practice, arrangement, and method will enable any one, professional or not, to perform this very useful minor operation with neatness, efficacy, and dispatch.

GALEN.

